# Exercise and reproductive function in polycystic ovary syndrome: protocol of a systematic review

**DOI:** 10.1186/s13643-017-0666-5

**Published:** 2017-12-22

**Authors:** Isis Kelly dos Santos, Romilson de Lima Nunes, Gustavo Mafaldo Soares, Tecia Maria de Oliveira Maranhão, Paulo Moreira Silva Dantas

**Affiliations:** 1 Postgraduate Program in Health Sciences (PPGCSA), Federal University of Rio Grande do Norte (UFRN), Natal, Brazil; 2 Department of Toco-Gynecology, Federal University of Rio Grande do Norte (UFRN), Natal, Brazil; 3 Department of Physical Education, Federal University of Rio Grande do Norte (UFRN), Natal, Brazil; 4 Postgraduate Program in Health Sciences (PPGCSA), Federal University of Rio Grande do Norte (UFRN), Natal, Brazil

**Keywords:** Exercise training, Menstrual cycle, Infertility, Polycystic ovary syndrome, Systematic review, Protocol

## Abstract

**Background:**

Although many post-participation outcomes in different types of physical training (e.g., aerobic and strength) have been previously investigated for the treatment of polycystic ovary syndrome, there is no recent systematic review of the relationship between various types of intervention and the reproductive function of women with PCOS. The current paper describes a systematic review protocol on the benefits of physical exercise and dietary or drug interventions on endocrinological outcomes in women with PCOS.

**Methods:**

PubMed/MEDLINE, Science Direct, Bireme, Scopus, Web of Science, ProQuest, Cochrane Library (Cochrane Systematic Reviews Database, Cochrane Central Register of Controlled Studies (CENTRAL) databases will be searched. Studies randomized controlled trials reporting on intervening changes in exercise interventions with or without interventions compared such as diet, medication and acupuncture on the menstrual cycle, and fertility in women with PCOS will be included. Results will be on the decrease of the characteristics of hyperandrogenism, insulin resistance, and obesity. Studies published since 2010 and in the English language will be included.

**Discussion:**

This systematic review will identify improvement strategies and types of interventions that are geared toward improving endocrine and consequently metabolic parameters. Thus, the use of such strategies may increase the types of low-cost non-drug therapies that aid in the treatment of PCOS.

**Systematic review registration:**

PROSPERO CRD42017058869

**Electronic supplementary material:**

The online version of this article (10.1186/s13643-017-0666-5) contains supplementary material, which is available to authorized users.

## Background

Polycystic ovarian syndrome (PCOS) is an endocrinopathy that affects women of reproductive age, whose main clinical features include menstrual dysfunction, infertility, clinical, and biochemical hyperandrogenism [1, 2]. Since these characteristics are the main concerns of patients with PCOS, and several therapeutic strategies are used in attempts to improve ovulatory function, several studies have shown that body weight loss can restore menstrual cycle and ovulation in PCOS women, an important factor to be verified and considered for the improvement of fertility in these patients [3–5].

Thus, lifestyle modification including exercise and diet has been recommended as the first-line therapy for women with PCOS, since this practice would reduce insulin resistance, improving metabolism and reproductive function [6, 7]. In addition, evidence-based guidelines recommend lifestyle modifications as non-pharmacological treatment for PCOS [8]. This article describes the protocol for a systematic review on the impact of physical exercise interventions on improving menstrual cycles and fertility (reproductive function) in women suffering from polycystic ovary syndrome.

## Methods/design

This systematic review protocol is reported according to the Preferred Reporting Items for Systematic Reviews and Meta-Analysis Protocols (PRISMA-P) 2015) statement [9] (see checklist Additional file 1) and developed with inspiration from the Cochrane Handbook for Systematic Reviews of Interventions [10]. This protocol was registered with the PROSPERO database (CRD42017058869).

The design of the present study favors the performance of the analyses, since when evaluating the effect of an intervention in the course of a clinical situation the randomized clinical trials are gold standard for the analysis of therapeutic interventions, allowing elimination of several biases in the intervention and control groups. In addition, this review can help the community in decision-making, on what methodologies and interventions to address with the target population, in search of significant results for the quality of life of those involved.

### Eligibility criteria

#### Inclusion criteria

Study design and participants: randomized controlled trials (randomized clinical trials in groups) on strategies (exercise with or without accompanying other interventions) to improve the reproductive function of women (18–40 years) diagnosed with PCOS.

Study configuration: clinical studies based on the implantation of the exercise as the main intervention, indicating cause and effect in the parameters of the reproductive function (changes in the hormonal levels responsible for the menstrual cycle, increase and improvement in the fertility (primary), decrease in the characteristics of hyperandrogenism, resistance to insulin, and obesity (secondary).The menstrual cycle and fertility should be specifically identified as the main outcome measure to be included in the analysis). In addition, studies must present interventions lasting more than 2 weeks of physical exercise, along with any other intervention (diet or medical treatment). Evaluation of at least one parameter or hormone of the reproductive function after the end of the exercise (post-test).

Period of time: studies published between 1 January 2010 and 31 December 2017 in the selected databases.

Language: English language.

For studies with duplicate results from the same study, only the most comprehensive and up-to-date version will be considered.

### Exclusion criteria

#### Interventional studies

Studies lacking prevalence rates, predictors, and/or pregnancy outcomes in adolescence with no data to calculate relevant effect sizes will be excluded from the quantitative analysis if the necessary information is not yet accessible even after several authors’ requests.

Unpublished manuscripts and conference summaries, as well as editorials, comments, reviews, letters to editors, and any publication without primary data.

### Information sources and literature search

Literature research strategies will be developed using indexed keywords in the Medical Subject Sections (MeSH), based on a combination of three search parameters: independent variable (exercise), dependent variable (menstrual cycle and infertility), and PCOS interest of the population). Further research will be done on the references of the selected articles. Studies published in the last 7 years (i.e., since 2010) will be included, this period was selected to ensure the relevance of the findings in the current health context, as well as the feasibility of further revisions on the subject. They will be identified in the following electronic databases: PubMed/MEDLINE, Science Direct, Bireme, Scopus, Web of Science, ProQuest, Cochrane Systematic Reviews Database, Cochrane Central Register of Controlled Studies (CENTRAL) can be found in Additional file 2. In addition to electronic databases, gray literature (i.e., unpublished and difficult to locate material) will be searched. Unpublished material will be identified by also searching the dissertation and thesis database, such as searching for relevant abstracts and contacts with authors to ensure that all relevant data is obtained. An experienced information specialist (IKS) will carry out all bibliographic searches. A preliminary systematic search strategy will be tested in order to obtain sufficient specificity, while maintaining high sensitivity, in which the Boolean operators AND and OR will be used. The research strategy contains components: exercise, menstrual cycle and/or infertility and/or hyperandrogenism and PCOS, through the relevant MESH terms, in Table 1.

**Table 1:** Search strategies

Search steps: exercise	Search steps: reproductive function	Search steps: PCOS
Sport	Ovulation	Polycystic ovary syndrome
Fitness	Hyperandrogenic chronic anovulation	Polycystic ovarian syndrome
Exercise therapy	Follicle-stimulating hormone (FSH)	Stein-Leventhal syndrome
Exercise training	Luteinizing hormone (LH)	
Exercise program	Menstrual Cycle	
Exercise regime	Menstruation; follicular phase	
Physical activity	Luteal phase	
Vigorous activity	Infertility	
Moderate activity	Female infertility	
Aerobic exercise	Sterility	
Aerobic capacity	Female sub-fertility	
Aerobic training		
Resistance training		

### Study selection process

In order to increase the reliability of screening studies and data among reviewers, a pilot test of a pre-defined screening form will be conducted based on the eligibility criteria. After searching the electronic databases, all studies identified in the search strategy are stored in a citation management system (EndNote) for the removal of the duplicates. Then, the titles and summaries of identified articles were independently selected by two authors in relation to the eligibility criteria, with any disagreement resolved by a third reviewer (i.e., level 1 screening). The full text of the articles and their potentially relevant references are acquired and selected to determine the final inclusion (i.e., selection of 2). Both reviewers should determine an outcome measure in the domains of interest indicated to verify the appropriateness of the studies. As the discrepancies encountered are resolved by consensus meetings or by a third reviewer, all information on the phases of the selection process will be identified in Table 2, based on the PRISMA Statement for Systematic Analysis Reports and Meta-Analysis Studies Health Care Interventions: Explanation and Drafting [11].

**Table 2:** Extraction and sorting of data

Information	Phases of a systematic review
Identification	Records identified through database search
	Additional records identified through other sources
	Records after duplicates removed
Screening	Records screened
	Records excluded
Eligibility	Full-text articles assessed for eligibility
	Full-text articles excluded, with reasons
Included	Studies included in qualitative synthesis
	Studies included in quantitative synthesis (meta-analysis)

### Data items and data collection process

Two reviewers of the research team will be responsible for independently extracting all pre-defined data for data extraction, if discordant information is observed, a third evaluator will participate in the data extraction and quality assessment part in order to summarize the main findings for final report. The data extraction table is available in Table 3. As in the study selection process, a standardized and modified pilot form will be tested for data extraction. Data extraction tables will be compared and integrated to ensure that all major findings are included and accurately in the review.

**Table 3:** Data extraction table

Data to be extracted	Item
Publication ID	Author
	Publication date
	Country of study
Study aim	Title/purpose/aim
Study design	Study type: randomized controlled trials, including randomized clinical trials in groups.
	Measurement tools, instruments, measures, outcome criteria
Sample characteristics	Number of participants
	Age
	Diagnostic criteria
Findings	Reported efficacy outcomes
	Reported side effects/menstrual cycles and fertility (reproductive function)
	Reported efficacy outcomes/(HOMA-IR, fasting Insulin, HDL, LDL, LDL), anthropometric variables (percentage of fat, lean mass, waist measurement, (TST, FAI, AMH, SHBG, DHEAS, LH, FSH ET)
	Reported health-related quality of life
	Any other findings
Strengths and limitations	Findings of critical appraisal

### Methodological quality/risk of bias appraisal

To evaluate the methodological quality of the studies, a standardized quality assessment tool for randomized control trials (RCT), the EPOC Risk of Bias Tool [12], will be used, and as a reference the check-list Preferred Reporting Items for Systematic Reviews and Meta-Analyses (PRISMA) [11].

### Synthesis of included studies

The results of the systematic review will be summarized descriptively, structured around the type of intervention, characteristics of the target population, type of outcome, and content of the intervention. The data from the intervention effect summaries for each study will be provided. When the studies use the same type of intervention and comparator with the same result, the results will be pooled using a random effects meta-analysis, with standardized mean differences for continuous results and risk ratios for binary results, and calculate double intervals 95% confidence interval and *P* values for each outcome.

In studies where the effects of grouping are not taken into account, an adjustment and standard deviations will be made for the design effect. The heterogeneity between the studies in measures of effect will be evaluated. We will perform sensitivity analyses based on study quality. We will use stratified meta-analyses to explore heterogeneity in effect estimates according to study quality, populations studied, the logistics of intervention, and intervention content. We will also evaluate evidence of publication bias through the evaluation, development and evaluation qualifications framework (GRADE) to assess the quality of the evidence set [13].

## Discussion

This protocol will lead to a systematic review of the impact of strategies to improve reproductive function in women with PCOS. At the end of the review, a solid end-of-project knowledge translation strategy will be implemented. The results of the systematic review will be presented at relevant meetings, either locally or nationally and internationally, and published in a peer-reviewed open access opinion journal to make the results accessible to the appropriate scientific and clinical public. The findings will also be disseminated through the newsletters (printed and online) in places of possible access of the target population. Knowledge and application of such strategies to improve reproductive function may reduce inappropriate costs of using health care, such as readmission to patients with acute care.

## Additional files

Additional file 1: PRISMA–P 2015 Checklist. (PDF 338 kb)

Additional file 2: Search strategy for PUBMED. (PDF 8 kb)

## Abbreviations

AMH: Antimüllarian hormone
DHEAS: Dehydroepiandrosterone sulfate
FAI: Free Androgen Index
FSH: Follicle-stimulating hormone
HbA_1c_: Hemoglobin A1c
HOMA-IR: Homoeostatic assessment of insulin resistance
IMC: Body mass index
LH: Luteinizing hormone
MeSH: Medical Subject Headings
PRISMA: Preferred Reporting Items for Systematic Reviews and Meta-Analyses
PRISMA-P: Preferred Reporting Items for Systematic Reviews and Meta-Analyses Protocols
RCT: Randomized controlled trials
SHBG: Sex-hormone binding globulin
PCOS: Polycystic ovary syndrome
T: Testosterone
VO_2 máx_: Maximum oxygen uptake
