# Supplementary material for: Exercise and reproductive function in polycystic ovary syndrome: protocol of a systematic review
Source: Syst Rev. 2017 Dec 22;6:264. doi: 10.1186/s13643-017-0666-5 (PMC5741916; doi:10.1186/s13643-017-0666-5)
Supplement: Supplementary file 2 — Search strategy for PUBMED. (PDF 8 kb) [file 13643_2017_666_MOESM2_ESM.pdf]

Database: PUBMED>
Search Strategy:
--------------------------------------------------------------------------------
1 Exercise
2 Sport
3 Fitness
4 Exercise therapy
5 Exercise training
6 Exercise program
7 Exercise regime
8 Physical activity
9 Vigorous activity
10 Moderate activity
11 Aerobic exercise
12 Aerobic capacity
13 Aerobic training
14 Resistance training
15 Ovulation
16 Follicle-stimulating hormone (FSH)
17 Luteinizing hormone (LH)
18 Menstrual Cycle
19 Menstruation
20 Follicular Phase
21 Luteal Phase
22 Infertility
23 Female Infertility
24 Sterility
25 Female Sub-Fertility
26 Polycystic ovary syndrome
27 Stein-Leventhal Syndrome
28 Function reproductive
29 Randomized control trials (RCT)
30 Clinical trial
31 Controlled clinical trial
32 Pragmatic Clinical Trial
